# Advanced Reinforcement Learning and Its Connections with Brain Neuroscience

**DOI:** 10.34133/research.0064

**Published:** 2023-03-15

**Authors:** Chaoqiong Fan, Li Yao, Jiacai Zhang, Zonglei Zhen, Xia Wu

**Affiliations:** ^1^ School of Artificial Intelligence, Beijing Normal University, Beijing, China.; ^2^ Faculty of Psychology, Beijing Normal University, Beijing, China.

## Abstract

In recent years, brain science and neuroscience have greatly propelled the innovation of computer science. In particular, knowledge from the neurobiology and neuropsychology of the brain revolutionized the development of reinforcement learning (RL) by providing novel interpretable mechanisms of how the brain achieves intelligent and efficient decision making. Triggered by this, there has been a boom in research about advanced RL algorithms that are built upon the inspirations of brain neuroscience. In this work, to further strengthen the bidirectional link between the 2 communities and especially promote the research on modern RL technology, we provide a comprehensive survey of recent advances in the area of brain-inspired/related RL algorithms. We start with basis theories of RL, and present a concise introduction to brain neuroscience related to RL. Then, we classify these advanced RL methodologies into 3 categories according to different connections of the brain, i.e., micro-neural activity, macro-brain structure, and cognitive function. Each category is further surveyed by presenting several modern RL algorithms along with their mathematical models, correlations with the brain, and open issues. Finally, we introduce several important applications of RL algorithms, followed by the discussions of challenges and opportunities for future research.

## Introduction

As an important subfield of machine learning and a promising solution to artificial intelligence (AI), reinforcement learning (RL) encompasses a wide variety of interactive learning approaches, which enables an agent to maximize a long-term reward function and to progressively understand an environment model based on experiences obtained by interacting with the unknown environment [1]. Owing to the feature that no prior knowledge is required and the outstanding decision-making performance, RL has proven its worth and found wide applications in a series of artificial domains, thus attracting widespread attention in the past decades and no doubt in the future. Despite the great potential, some tough challenges remain open, such as enhancing learning efficiency from limited experiences, balancing efficiency and flexibility, adapting to environmental changes, generalizing learning from a small number of tasks, scaling to high dimensions and continuous spaces, interacting among multiple agents, and so on [2].

Seeking inspirations from brain neuroscience is one of the most promising solutions to achieve human-level or even superhuman intelligence, and thus has become an intriguing area of AI [3]. The relationship between RL in computer science and decision mechanism in the brain has been gradually revealed by Niv [4] and Ludvig et al. [5]. These preliminary studies indicate the fact that the human brain possesses an innate capability to solve the above-mentioned issues and even beyond [6]. Those findings and evidences fuel a resurgence of interest in RL, and lead to confidence regarding the development of brain neuroscience-inspired solution toward efficient, flexible, adaptive, generalizable, scalable, and collaborative RL approaches.

Thanks to the development of brain neuroscience at the biological and psychological levels, the past years have witnessed a rapid rise of the brain-inspired/related RL algorithms that mimic the way our brain works to accelerate learning efficiency and improve the performance of decision making. To provide a comprehensive perspective, several survey papers in this field have been introduced [7–9]. Specifically, Neftci and Averbeck [7] described RL in artificial and biological systems, and presented learning to learn (meta RL), model-based RL, and hierarchical RL. Subramanian et al. [8] revealed a number of findings in neuroscience that provided evidences for the plausibility of RL and briefly reviewed several RL algorithms including distributional RL, meta RL, hierarchical RL, and so on. Unfortunately, the 2 works just list some modern RL algorithms that are related to the brain without providing a proper classification and fail to clarify the detailed relationships with brain mechanism. Eckstein et al. [9] focused on the interpretability and generalizability of RL, and clarified the connections and differences of RL between machine learning, psychology, and neuroscience.

Different from previous works, in this paper, we present a more comprehensive introduction to the brain neuroscience-inspired/related RL algorithms. We classify them into 3 categories according to their connections to the brain. It is worth noting that the neural network-based RL, known as deep RL, is relatively mature and more popular compared with the other advanced RL schemes presented in the rest of this paper. Besides, there are already some review articles that comprehensively introduce the deep RL, e.g., refs. [10–12]. Therefore, we do not specifically introduce the original deep RL in the following presented advanced RL. Certainly, the advanced brain neuroscience-inspired RL algorithms can be easily extended to deep RL algorithms by applying deep neural networks as functional approximations, and some frontier research that will be introduced in the following parts has applied brain neuroscience into the original deep RL to achieve the brain neuroscience-related deep RL [13]. It is expected that this work can contribute to bridging the gap between RL and brain neuroscience, and can boost the research of advanced RL that built upon inspirations from brain neuroscience. The main contributions of this work are listed as follows.

• To facilitate the understanding of advanced RL algorithms and their connections with the brain, we first introduce the preliminaries of RL and brain neuroscience. Specifically, for RL, with the basic framework and some essential concepts, 2 optimization schemes (i.e., value-based and policy-based) along with their classical algorithms are provided. For brain neuroscience, by presenting the brain anatomy, we clarify the main regions involved in RL and describe the neural representations of RL elements.

• After providing introductions to RL and brain neuroscience, we comprehensively investigate the increasingly popular RL algorithms that are related to brain mechanism. We classify them into 3 categories according to different inspirations from brain neuroscience, i.e., micro-neural activity, macro-brain structure, and cognitive function. Specifically, in the first category, we introduce distributional RL, stigmergy RL, and successor representation (SR) RL. In the second category, we present hierarchical RL, meta RL, prefrontal RL, and an RL algorithm inspired by the interactions between 2 brain regions. In the third category, we introduce attentional RL and episodic RL, and then briefly mention other RL algorithms that are related to the cognitive function. For each algorithm, we describe its mathematical model, connections with the brain, and open issues.

• For practical applications of RL algorithms, we introduce a range of fields including game, robotics, natural language processing (NLP), and computer vision (CV). Then, we discuss the challenges and opportunities of this amazing interdisciplinary field. It is realized that only through multifield cooperation can brain-like intelligence be realized.

**Fig. 1.**
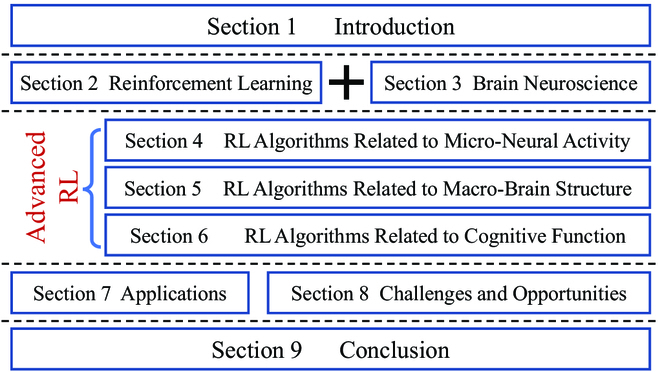
Organization of this paper.

The remainder of this paper is organized as follows. We introduce the related preliminaries of RL and brain neuroscience in the “Reinforcement Learning” and “Brain Neuroscience” sections, respectively. Advanced RL schemes are comprehensively described in the following 3 sections, with the “RL Algorithms Related to Micro-Neural Activity” section for micro-neural activity-inspired/related RL algorithms, the “RL Algorithms Related to Macro-Brain Structure” section for macro-brain structure-inspired/related RL algorithms, and the “RL Algorithms Related to Cognitive Function” section for cognitive function-inspired/related RL algorithms. We list some typical applications in the “Applications” section, and discuss the challenges and opportunities for the research on the brain-inspired/related RL field in the “Challenges and Opportunities” section. Finally, the conclusion is drawn in the “Conclusion” section. For the sake of clarity, the organization of this paper is shown in Fig. 1.

## Reinforcement Learning

**Fig. 2.**
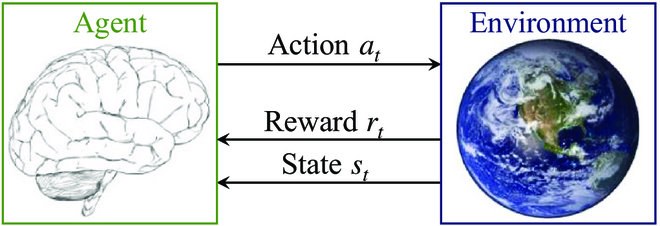
Interaction between agent and environment.

The nature of RL is learning through interaction. As shown in Fig. 2, an RL agent interacts with a dynamic environment through taking actions under different states and learns to update its choice according to the correspondence rewards. This learning paradigm has its rationality in behaviorist psychology and is a main theoretical foundation of RL [1]. In the following, we first introduce Markov decision process (MDP) and the optimization goals. Then, with some necessary concepts, 2 main RL paradigms, i.e., value optimization and policy optimization, are presented. Table 1 lists the mentioned RL algorithms and some references.

**Table 1.** Summary of the following mentioned RL algorithms.

Type	Algorithm	References
Value optimization	SARSA	[1,99]
	*Q*-learning	[100–102]
Policy optimization	REINFORCE	[17,18]
Hybrid optimization	Actor–critic	[103–105]

### Markov decision process

MDP is a mathematical framework to model decision-making problems, where the state of any decision does not depend on the history of the previous states, but instead depends only on the current state and the adopted action of an agent [14]. Typically, an MDP is defined by a quadruple $\langle S,\;A,\;T,\;R\rangle$, where $S=\left\{s\right\}$ is a set of all possible states, $A=\left\{a\right\}$ is a set of all possible actions, $T:S\times A\times S\to \left[0,1\right]$ is the state transition probability function that maps a state–action pair at time *t* into a distribution of states at time *t* + 1 with *T*(*s_t_*, *a_t_*, *s*_*t* + 1_) =  Pr (*s*_*t* + 1_| *s_t_*, *a_t_*), and R:S×A×S→ℝ is the reward function with *r_t_*(*s_t_*, *a_t_*, *s*_*t* + 1_) representing the instantaneous reward that the agent receives when action *a_t_* leads from state *s_t_* to state *s*_*t* + 1_.

As mentioned above, in an environment state *s*, the agent needs to select an action *a* from the actions set A. Policy *π* is used to describe this action selection preference, which is a mapping from states to a probability distribution over actions, i.e., π:S×A with *π*(*a*| *s*) =  Pr (*a_t_* = *a*| *s_t_* = *s*) representing the probability of selecting an action a∈A in a state s∈S. The objective of MDP formulation is to achieve an optimal policy *π*^∗^ to maximize the reward. MDP problems could be finite or infinite in time horizon. For MDPs with finite time horizon, to maximize the expected total reward, the optimal policy *π*^∗^ is defined as:

$$\begin{array}{c} \pi^{*}=\arg\;\max_{\pi }\;E_{\pi }\left[\sum_{t=0}^{T}r_{t}\left(s_{t},\;\pi \left(a|s_{t}\right)\right)\right]. \end{array}$$ (1)

For MDPs with an infinite time horizon, the expected discounted total reward is considered, and hence the optimal policy *π*^∗^ is given by:

$$\begin{array}{c} \pi^{*}=\arg\;\max_{\pi }\;E_{\pi }\left[\sum_{t=0}^{T}\gamma^{t}r_{t}\left(s_{t},\;\pi \left(a|s_{t}\right)\right)\right], \end{array}$$ (2)

where *γ* ∈ [0, 1] is the discount factor to determine the importance of future reward compared with the current one. Specifically, if *γ* → 0, the agent would be more interested in its current reward; if *γ* → 1, the agent will strive for a long-term reward.

### Value optimization

Methods based on value optimization aim to obtain an optimal value function through dynamic programming. Hereafter, we explain the concepts of value function and dynamic programming, with which 2 representative temporal difference (TD) learning algorithms are presented.

#### Value function

A value function is a prediction of the expected discounted total reward, measuring how good each state, or state–action pair, is. The state-value function *V^π^*(*s*), referred to as the value of a state, is the expected return given an agent starts in state *s* and follows policy *π* thereafter, i.e.,

$$V^{\pi }\left(s\right)=E_{\pi }\left[\sum_{k=0}^{\infty }‍\gamma^{k}r_{t+k}|s_{t}=s\right].$$ (3)

Similarly, the action-value function *Q^π^*(*s*, *a*), representing the value of a state–action pair, is the expected utility after taking action *a* in state *s* with policy *π*, i.e.,

$$Q^{\pi }\left(s,a\right)=E_{\pi }\left[\sum_{k=0}^{\infty }‍\gamma^{k}r_{t+k}|s_{t}=s,a_{t}=a\right].$$ (4)

Then, according to the Bellman equation, the state-value function *V^π^*(*s*) and the action-value function *Q^π^*(*s*, *a*) can decompose into the following forms:

$$\begin{array}{c} \left\{\begin{array}{c} V^{\pi }\left(s\right)=E_{\pi }\left[r_{t}\left(s,\;a\right)+\gamma V^{\pi }\left(s_{t+1}\right)\right],\\ Q^{\pi }\left(s,\;a\right)=E_{\pi }\left[r_{t}\left(s,\;a\right)+\gamma Q^{\pi }\left(s_{t+1},\;\pi \left(a|s_{t+1}\right)\right)\right]. \end{array}\right. \end{array}$$ (5)

An optimal state-value *V*^∗^(*s*) is the maximal state-value achieved by any policy for state *s*, and an optimal action value *Q*^∗^(*s*, *a*) is the maximal action value achieved by any policy for state *s* with action *a*. Besides, according to the recursive forms of *V*^∗^(*s*) and *Q*^∗^(*s*, *a*) in Eq. 5, we have:

$$\begin{array}{c} \left\{\begin{array}{c} V^{*}\left(s\right)=\max_{\pi }V^{\pi }\left(s\right)=\max_{a\in A}\left\{E_{\pi }\left[r_{t}\left(s,\;a\right)+\gamma V^{*}\left(s^{\prime }\right)\right]\right\},\\ Q^{*}\left(s,\;a\right)=\max_{\pi }Q^{\pi }\left(s,\;a\right)=r_{t}\left(s,\;a\right)+E_{\pi }\left[\max_{a\prime \in A}\gamma Q^{*}\left(s^{\prime },\;a^{\prime }\right)\right]. \end{array}\right. \end{array}$$ (6)

#### Dynamic programming

Dynamic programming refers to a class of algorithms that can be used to find optimal policy with environment models, such as MDPs. Due to the perfect model assumption and the high computational expense, traditional dynamic programming algorithm is of limited application. However, it offers an essential foundation for the comprehension of RL, which releases the perfect assumption of environment model and reduces computational complexity. The key idea of dynamic programming, and of RL generally, is using value functions to guide the policy search process [15], which can be achieved by policy iteration and value iteration.

**Policy iteration**: Policy iteration alternately implements policy evaluation and policy improvement to generate a series of improving policies. In policy evaluation, the state-value function *V^π^* of the policy *π* is estimated, and in policy improvement, the state-value function *V^π^* is used to generate an improved policy *π*^′^, i.e.,

$$\pi_{0}\overset{E}{⟶}V^{\pi_{0}}\overset{I}{⟶}\pi_{1}\overset{E}{⟶}V^{\pi_{1}}\overset{I}{⟶}\pi_{2}\cdots \pi_{*}\overset{E}{⟶}V^{\pi_{*}},$$ (7)

where $\overset{E}{⟶}$ represents the process of policy evaluation and $\overset{I}{⟶}$ represents the process of policy improvement.

Unfortunately, since each round of iteration involves policy evaluation over the entire state set, the computation complexity of protracted iteration is extremely high, and the convergence holds only in the limit. To this end, value iteration is adopted.

**Value iteration**: Value iteration is a scheme that truncates the policy evaluation steps after just one sweep. It can be expressed as a simple backup operation that combines the policy improvement and the truncated policy evaluation, i.e.,

$$\begin{array}{c} V_{k+1}\left(s\right)=\underset{a\in A}{\text{max}}\;E_{\pi }\left[r_{t}+\gamma V_{k}\left(s^{\prime }\right)\right],s\in S. \end{array}$$ (8)

For any *V*_0_, the sequence {*V_k_*} converges to an optimal state value *V*^∗^ under the condition that *V*^∗^ exists.

#### Temporal difference learning

As a core idea of RL, TD learning is a combination of Monte Carlo methods and dynamic programming methods [1]. It is similar to Monte Carlo in that it can learn directly from historical experience without a model of the environment’s dynamics, and similar to dynamic programming, the parameters can be updated flexibly in the learning episode. TD is a model-free RL approach learning state-value function *V*(*s*) with the TD error *δ_t_*,

$$\delta_{t}=r_{t}+\gamma V\left(s_{t+1}\right)-V\left(s_{t}\right),$$ (9)

which describes the difference between a real transition and the expectation. Then, the value function is updated as:

$$V\left(s_{t}\right)=V\left(s_{t}\right)+\alpha \delta_{t},$$ (10)

where *α* is a learning rate. Precisely, Eq. 10 denotes the simplest tabular TD(0) learning, where “0" means one-step returns.

Two typical TD learning mechanisms for value function evaluation are presented, i.e., the on-policy algorithm SARSA, and the off-policy algorithm *Q*-learning.

**SARSA**: As an on-policy method, it requires to estimate *Q^π^*(*s*, *a*) for the current policy *π* and for all states *s* and actions *a*. SARSA (an abbreviation of state, action, reward, next state, and next action) uses a quintuple (*s_t_*, *a_t_*, *r_t_*, *s*_*t* + 1_, *a*_*t* + 1_) to constitute a transition from the current state–action pair to the next state–action pair. The update rule of SARSA is given by:

$$Q\left(s_{t},a_{t}\right)⟵Q\left(s_{t},a_{t}\right)+\alpha \left[r_{t}+\gamma Q\left(s_{t+1},a_{t+1}\right)-Q\left(s_{t},a_{t}\right)\right].$$ (11)

***Q*-learning**: An amazing breakthrough in RL is the development of an off-policy TD learning approach, known as *Q*-learning. *Q*-learning optimizes the policy greedily with respect to action-value function via the max operator. The update rule of one-step *Q*-learning is given by:

$$\begin{array}{c} Q\left(s_{t},\;a_{t}\right)⟵Q\left(s_{t},\;a_{t}\right)+\alpha \left[r_{t}+\gamma \max_{a}Q\left(s_{t+1},\;a\right)-Q\left(s_{t},\;a_{t}\right)\right]. \end{array}$$ (12)

### Policy optimization

In contrast to methods based on value optimization, policy optimization methods do not learn value function; instead, they try to find the optimal policy that receives the most accumulated utility via directly searching the policy space. That is, a policy optimization algorithm assesses the performance of a candidate policy by comparing the overall reward achieved in one or several episodes following that policy. Compared with value optimization-based methods, policy optimization-based methods can perform well in scenarios with continuous action spaces. However, due to the high variance in the gradient estimation, the learning efficiency of policy optimization would be relatively low.

#### Policy gradient

Since gradients contain a strong signal to instruct the improvement of a parameterized policy *π*(*a*| *s*; ***θ***), policy gradient is a typical algorithm in RL. Policies could be deterministic or stochastic. The deterministic policy gradient for decision-making problems with continuous action space is introduced in Silver et al. [16], which is represented by the expected gradient of the action-value function, while the stochastic policy gradient integrates both state and action spaces, and calculates the expected gradient of the state-value function and the action-value function. Thus, the deterministic policy gradient would be easier to estimate than the stochastic policy gradient.

For a differentiable policy *π*(*a*| *s*; ***θ***), the gradient can be computed analytically. When it is nonzero, it holds that

$$\begin{array}{c} \nabla_{\theta }\pi \left(a|s;\theta \right)=\pi \left(a|s;\theta \right)\frac{\nabla_{\theta }\pi \left(a|s;\theta \right)}{\pi \left(a|s;\theta \right)}=\pi \left(a|s;\theta \right)\nabla_{\theta }\log\pi \left(a|s;\theta \right), \end{array}$$ (13)

where ∇_***θ***_ log *π*(*a*| *s*; ***θ***) is called a likelihood ratio. According to the policy gradient theorem [17], for a differentiable policy *π*(*a*| *s*; ***θ***), the policy gradient is given by:

$$\begin{array}{c} \begin{array}{l} \nabla J\left(\theta \right)=E_{\pi }\left[\sum_{a}Q^{\pi }\left(s_{t},\;a\right)\nabla_{\theta }\;\pi \left(a|s_{t};\theta \right)\right]\\ =E_{\pi }\left[\sum_{a}\pi \left(a|s_{t};\theta \right)Q^{\pi }\left(s_{t},\;a\right)\nabla_{\theta }\log\pi \left(a|s_{t};\theta \right)\right]\\ =E_{\pi }\left[Q^{\pi }\left(s_{t},\;a_{t}\right)\nabla_{\theta }\log\pi \left(a_{t}|s_{t};\theta \right)\right]. \end{array} \end{array}$$ (14)

REINFORCE [18] is a typical policy gradient algorithm. By adopting return *r_t_* as an unbiased sample of *Q*(*s_t_*, *a_t_*), REINFORCE updates the parameter ***θ*** as follows:

$$\theta_{t+1}≐\theta_{t}+\alpha r_{t}\nabla_{\theta }\log\pi \left(a_{t}|s_{t};\theta_{t}\right),$$ (15)

where vector *r_t_*∇_***θ***_ log *π*(*a_t_*| *s_t_*; ***θ****_t_*) is the direction in the parameter space that maximizes the probability of repeatedly selecting the action *a_t_* in the state *s_t_*.

To reduce the variance of gradient estimation, a baseline *b_t_*(*s_t_*) is usually subtracted from the return, and the gradient direction is yielded via keeping the unbiasedness. In this case, the policy gradient can be expressed as:

$$\begin{array}{c} \nabla J\left(\theta \right)\propto \nabla_{\theta }\log\pi \left(a_{t}|s_{t};\theta_{t}\right)\left[Q\left(s_{t},\;a_{t}\right)-b_{t}\left(s_{t}\right)\right]. \end{array}$$ (16)

Thus, a new gradient updating form of REINFORCE that contains baseline is given by:

$$\begin{array}{c} \theta_{t+1}≐\theta_{t}+\alpha \left[r_{t}-b_{t}\left(s_{t}\right)\right]\nabla_{\theta }\log\;\pi \left(a_{t}|s_{t};\theta_{t}\right), \end{array}$$ (17)

which is a generalization of Eq. 15.

#### Actor–critic

Actor–critic is an integration of value-function mechanism and policy-gradient mechanism, where the critic learns a parameterized value function and the actor learns a parameterized policy in the direction suggested by the critic. Therefore, the actor–critic algorithm combines the advantages of both value-function and policy-gradient schemes; i.e., the actor can manage continuous action spaces, and the critic accelerates the learning procedure by reducing variance of gradients. Note that, although the above RL with baseline introduction also learns both value function and policy function, it is not regarded as an actor–critic, since its value function is just as a baseline, and is not used for bootstrapping.

The related parameters of value function and policy function are updated according to the TD error *δ_t_*. For the one-step actor–critic method, the parameter ***θ*** is updated as:

$$\theta_{t+1}≐\theta_{t}+\alpha \delta_{t}\nabla_{\theta }\log\;\pi \left(a_{t}|s_{t};\theta_{t}\right).$$ (18)

Compared with value optimization-based methods, actor–critic approaches usually have good convergence and can learn an explicitly stochastic policy, which would be useful for continuous-valued actions and non-Markov cases. In addition, due to the convenience of imposing domain-specific constraints on the set of available policies, the separate actor in actor–critic makes the method more appealing in some aspects such as physiological and psychological models.

## Brain Neuroscience

Recently, substantial progresses have been made in understanding some of the brain mechanisms and in linking the computational models with neurobiological and neuropsychological findings. To make brain anatomy and some relevant neurophysiologies more accessible to researchers in the field of machine learning and AI, we introduce some background knowledge of the brain neuroscience field associated with RL and reward-driven decision making in this section.

### Anatomy of the brain

A fundamental requirement for finding the intrinsic connection between RL and neuroscience is the knowledge of brain anatomy, which has been well studied in the field of neurobiology. Generally, the brain comprises 3 major parts, i.e., the cerebral hemispheres, the brainstem, and the cerebellum. The cerebral hemispheres can be further separated into 4 lobes: frontal, parietal, occipital, and temporal. Neurons in these lobes are mainly distributed in the cerebral cortex. They establish connections between regions of the cortex as well as neurons outside the cortex with synaptic transmission. Numerous clusters of neurons, named nuclei, are distributed all over the brainstem and the cerebral hemispheres, as well as their corresponding junctions.

The thalamus and especially the basal ganglia (BG) are 2 principal structures in the forebrain related to RL. It is widely believed that BG is in charge of control and movement, and is located on both sides of the upper brainstem. The BG contains numerous nuclei, which are generally divided into 2 types: the *striatum* and the *globus pallidus*, and they play a vital role in the process of neuromodulation. Specifically, the striatum receives inputs from the cerebral cortex and the multitudinous nuclei, while the globus pallidus is responsible for outputting feedback information from the BG to the cerebral cortex through the thalamus.

The neural circuitry between the BG and other brain regions is extremely complex, among which the most widely studied are the input paths from the ventral tegmental area (VTA) to the striatum. Neurons in these paths release modulatory dopamine. One major function of dopamine is to provide information about rewards, which, in turn, can mediate behavioral actions. Besides, the dopamine neurons originated from the VTA can project to many other regions of the brain in a diffuse manner, and thus also have effects on memory consolidation and mood state.

### Neural representations of RL elements

The connection between the theoretical framework of RL and the neural mechanism of the brain has been unveiled progressively [19]. Numerous brain regions are related to RL in different aspects, and substantial correlations between neural activities in brain regions and important components of RL model have been revealed gradually [20]. Here, we concentrate on the neural representations of 3 fundamental components in RL, i.e., TD error, value function, and action selection.

#### Representation of TD error

As mentioned in the “Reinforcement Learning” section, the TD learning algorithms minimize the TD errors to improve the accuracy of the *Q* values. The hallmark of TD errors is that they occur only when stimuli are unpredicted. In the field of computational neuroscience, a similar reward prediction error (RPE) has been found in dopamine neurons’ activities in many brain regions including VTA, midbrain, dorsolateral prefrontal cortex (PFC), anterior cingulate cortex (ACC), and striatum [21,22]. It is observed that dopamine neurons show burst discharge to unpredicted rewards, while after conditioning has been built, the neurons fire only in response to the conditioned stimulus, not to the reward [23]. The discharge phenomena of dopamine neurons in multiple brain areas perfectly match the characteristic of TD error in RL algorithms.

The close resemblance between the firing patterns of dopamine neurons and the RPE in TD algorithms motivates the RPF hypothesis of dopamine, which further catalyzes the subsequent extensive researches about the correspondence between RL algorithms in the computer science and reward-driven learning in the brain.

#### Representation of value function

As characterized by Eqs. 3 and 10, value function is the reward prediction from which the RPE is generated. Given the widespread projections of dopamine neurons, it is intuitive that neural signal carrying information about reward expectation would exist widely within the brain [24]. This view finds support from the fMRI studies in the brain regions of the ventral striatum and PFC.

Similar to the value function approximation in RL, it is revealed that the brain also uses state-value function and action-value function encoding [25]. Specifically, neural signals related to state-value functions are detected in the ventral striatum, ACC, and amygdala. They play more evaluative roles for all the alternative choices, while neural signals related to action-value functions are stored and updated at the synapses between cortical axons and striatal spiny dendrites. They are useful in selecting a particular action especially before and during a motor response. Unsurprisingly, transformations between the 2 types of value function have been demonstrated in the brain. For example, during the decision-making period, the state value would change from the average action values of all possible choices to the action value of the selected one.

In addition, neural signals of selected action values that correspond to after-decision state values have been found in the orbitofrontal cortex, medial frontal cortex, dorsolateral PFC (dlPFC), and striatum. The fact that neural signals associated to value function are universal within the brain indicates that they possibly contribute not only RL approach, but also other cognition-inspired schemes.

#### Representation of action section

In the decision-making process, neural activities related to action-value function need to be transformed to the neural signals related to a specific action, and then be delivered to the region responsible for movement. Therefore, some brain regions encoding action-value functions are possibly involved in action selection [20]. Yet, the accurate area that plays important roles in action selection could be different for different behavioral tasks.

Besides, neurons in multiple brain regions responsible for motor control often display activity prior to specific movements, which indicates that they would be involved in action selection. Specifically, movements are closely related to neural activity in different brain areas, such as the premotor cortex, primary motor cortex, frontal eye field, supplementary eye field, posterior parietal cortex, and superior colliculus. These structures are tightly connected with motor control in the brainstem.

Apart from the brain areas involved in neural activities of action value and motor control, other regions such as the medial frontal cortex and the orbitofrontal cortex may have potential influences on the process of action selection [26]. Taking the medial frontal cortex for example, its neural signals related to an upcoming movement are more active than other areas of the brain.

### Summary

We present some important preliminaries in brain anatomy for understanding the neural foundations of RL, and introduce some major neuroscience findings that provide evidences for the connections between neural mechanisms in multiple brain areas and RL components. These influential results have encouraged research on brain-inspired RL schemes. It should be highlighted that those computational models of RL also have a transformational effect on the field of neuroscience, helping to detail what and how these neurons are working when humans make decisions.

## RL Algorithms Related to Micro-Neural Activity

In this section, we introduce 3 advanced RL algorithms that are related to the micro-neural activity of the brain, i.e., distributional RL, stigmergy RL, and SR RL. Besides, we present a summary of these RL algorithms including their micro-neural activity correlations, advantages, and relevant references in Table 2.

**Table 2.** Micro-neural activity-inspired/related RL algorithms.

Algorithm	Micro-neural activity correlations	Advantages	References
DRL	Dopamine neuron responses and prefrontal cortical responses	Learning the full reward distribution	[27–30]
SRL	Interaction between various synapses	Multi-agent collaboration with independent learning	[36,37]
SR-RL	Hippocampal neuron responses	Achieving trade-off between efficiency and flexibility	[38,39,106]

### Distributional RL

#### Description of the algorithm

In the classical TD learning, as discussed in the “Reinforcement Learning” section, the state value is represented as an expectation of future rewards starting from that state, which may not capture the high-order information of reward. To remedy this shortcoming, a novel framework for value-based RL, named distributional RL (DRL), which maintains an expected reward distribution instead of a single average value over long-term rewards, is developed. DRL focuses on updating a random variable *Z*(*s*, *a*), whose expectation is the action value *Q*(*s*, *a*), i.e., $Q\left(s,a\right)=E\left[Z\left(s,a\right)\right]$. It follows that

$$Z\left(s_{t},a_{t}\right)=r_{t}+\gamma Z\left(s_{t+1},a_{t+1}\right).$$ (19)

Recently, many studies have been conducted on DRL. As a fundamental work of DRL, Bellemare et al. [27] demonstrated some properties of the distributional Bellman operator and applied it to learn the approximate value distribution. With a different expression of distribution, Dabney et al. [28] designed a quantile regression-based DRL that adopts quantiles to describe the distribution. Taking the representation ability of DRL under limited samples into consideration, Yang et al. [29] developed a fully parameterized quantile function for DRL, which contains 2 networks, a fraction proposal network and a quantile value network. Despite the important contributions, one obvious disadvantage of these quantile regression-based DRLs is that the monotonicity of the quantile value cannot be guaranteed, which results in the failure to learn an effective distribution and deterioration in performance. To this end, by introducing a constraint of monotonicity when estimating the quantile value, a noncrossing quantile regression method was proposed in Zhou et al. [30].

#### Micro-neural activity correlations

DRL has shown to be biologically plausible for dopaminergic and cortical mechanisms. First, for dopamine neuron responses, via single-unit recordings from the mouse VTA area, Dabney et al. [31] showed that for a dopamine-related reward, different neurons carry different RPEs. The RPEs could be positive or negative for a reward, indicating a varying degree of optimism or pessimism for achieving a particular goal, respectively. Through extensive experiments, results show that compared with other models, DRL achieves the most accurate prediction of the RPE turning points and future rewards in the brain. In addition, PFC is also a strong candidate for DRL. With the findings of DRL in dopamine neurons, and the evidence that PFC (as a main recipient of dopamine input) encodes abundant reward-related signals such as learning rates and discount factor, DRL also interprets reward responses in many subregions of PFC, particularly the ACC [32].

Note that the framework of DRL was proposed independently in computer science before it has been used to explain the neural mechanism. Therefore, instead of being inspired by the brain, the idea of DRL in turn provides a more efficient computational model for brain exploration. Some of the following presented RL algorithms also hold the same position.

#### Open issues

The study of DRL remains largely open. First, there are still obvious approximation errors for modeling the reward distribution. Besides, the performance of DRL in the tasks with the exploration-critical feature is relatively inferior. How to construct a precise model of the reward distribution and how to employ the complete information of the reward distribution to improve the exploration ability are challenging problems to be solved. Future work can devote to identify neural architectures and mechanisms that may provide some inspirations for the enhancement of DRL.

### Stigmergy RL

#### Stigmergy mechanism

Stigmergy comprises 4 key elements: medium, trace, condition, and action, which together constitute a feedback loop between agents and the environment [33]. The medium is the information aggregator to enable multiagent collaboration. The trace is the digital pheromone left by agents in the medium to indicate the environmental change resulting from their actions, which can superposition, diffuse and decay with time. Then, these digital pheromones would influence the subsequent actions of other agents in a mutual way, and the degree of the influence mainly depends on the distance between agents.

#### Interaction between synapses

Recent experimental evidences in neuroscience indicate that, resembling the importance of glial cells in the central nervous system, astrocytes play a critical role in the regulation of synaptic transmission. Owing to the enrichment of various receptors, astrocytes can be involved in many neural modulations, and the interaction between synapses is mainly reconciled by the propagation of calcium ion within astrocytes. First, the synaptic state change caused by consecutive action potentials can create calcium elevations in the related domains. Then, the calcium elevations would diffuse into other domains, which constitute important interactions between diverse synapses and provide a feedback regulation [34]. It is also demonstrated that the synaptic transmission efficiency through the presynaptic terminal will be remarkably reduced without the calcium signal [35]. Therefore, astrocytes can be regarded as a spatial monitoring network, where synapses can interact with others through the inherent properties of calcium ion even though there is no direct neural connection between them.

It is revealed that the stigmergy mechanism has many similarities with the interactive activities between synapses in the brain [36]. Specifically, miscellaneous synapses can be regarded as different agents, a map of calcium ion consistence within astrocytes are the medium, and astrocytes are the medium carriers obviously. Besides, the process of calcium elevations triggered by synaptic state change corresponds to the leaving traces in the medium, and the regulation provided by astrocytes for synapses corresponds to the condition provided by the medium for agents.

#### Description of the algorithm

**Fig. 3.**
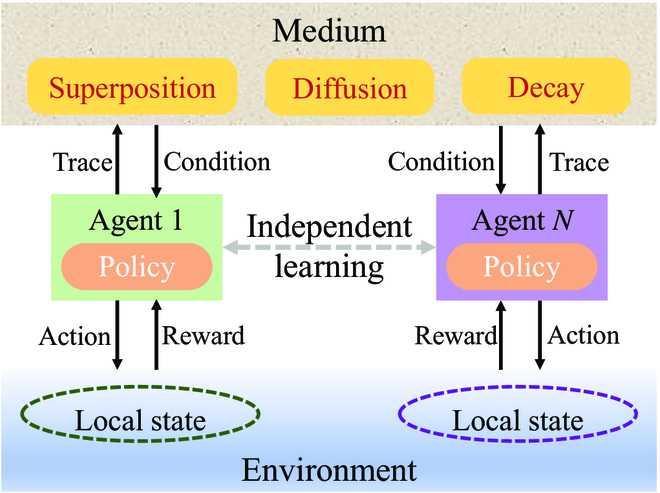
Framework of the stigmergy RL model.

According to this correspondence, a synapse interaction-inspired stigmergy RL (SRL) algorithm is proposed in Xu et al. [37], which is shown in Fig. 3. In particular, with partial and local observations of the environment, each agent has to learn and act independently. To enable effective collaboration, a stigmergy-based medium is deployed as an indirect communication bridge for the agents. When agents interact with the environment, they will leave trace (i.e., digital pheromone) in the medium, which can be denoted as some instructional records with information including value, time, and location. The medium (i.e., a map of digital pheromone) that carries the distribution of digital pheromone would return condition to guide the subsequent action selections of agents. The digital pheromone coming from different agents can superpose linearly, diffuse mutually, and decay over time. Therefore, the medium can be updated constantly by mutual communications among agents in the relevant area.

Denote the overall amount of digital pheromone in area A at time *t* as $\xi_{A}\left(t\right)$. In SRL, any agent would perform the action selection and simultaneously leave its digital pheromone in the medium to generate new information in the environment for the subsequent action. The updating rule of the digital pheromone $\xi_{A}\left(t\right)$ within this process is given by:

$$\begin{array}{c} \xi_{A}\left(t+1\right)=\left\{\begin{array}{c} \xi_{A}\left(t\right)+c_{1},\text{if}\;A\;\text{is the labeled area},\\ c_{2}\times \xi_{A}\left(t\right),\text{otherwise}, \end{array}\right. \end{array}$$ (20)

where *c*_1_ is the amount of digital pheromone left by an agent, and 0 < *c*_2_ < 1 is a discount constant.

### Successor representation RL

#### Description of the algorithm

In the past 2 decades, theories of RL have focused on 2 families of algorithms, known as model-based and model-free, which are distinguished by the used representations and computations for value estimation. Model-based algorithms learn the underlying “model" (i.e., the transition function and reward function) and use this model to compute an estimation of value functions by iterative computation, whereas model-free algorithms directly estimate the value function from the state–action reward sequence without learning the specific expression of the reward function and transition function. Therefore, the former is more flexible but less efficient, and the latter is contrary.

SR provides a third option for RL to learn value functions, which can intermediate the above 2 categories of RL algorithms and thereby balances the flexibility and efficiency. The basic idea of SR is to construct a “predictive map" of the environment that summarizes the long-range predictive relationships between states of the environment. Mathematically, the SR can be defined as an expectation of the discounted state occupancy, which is given by [38]:

$$\begin{array}{c} M\left(s,\;s^{\prime },\;a\right)=E\left[\sum_{t=0}^{\infty }\gamma^{t}1\left[s_{t}=s^{\prime }\right]|s_{0}=s,\;a_{0}=a\right], \end{array}$$ (21)

where 1[·] = 1 when the argument is true and zero otherwise.

Given the definition of SR, the state-value function can be decomposed into 2 parts, i.e., a reward predictor and a successor map. Thus, the *Q* value for choosing action *a* in state *s* can be expressed as the inner product between the SR and the instantaneous reward, i.e.,

$$Q^{\pi }\left(s,\;a\right)=\sum_{s^{\prime }\in S}‍M\left(s,\;s^{\prime },\;a\right)r\left(s^{\prime }\right).$$ (22)

#### Micro-neural activity correlations

Recent neural studies have provided evidences for the SR [39]; meanwhile, the SR has been used as a model for describing different biological phenomena. In the 2 directions, the hippocampal neuron is prominent. First, in the brain, supposing a collection of neurons encoding the spatial function for each state, the resulting population code will closely resemble classical place fields in the hippocampus. This confirms that the properties of rodent hippocampal place cells can be captured by the SR. Besides, for the tasks with a more abstract sequential structure, the similarity between the hippocampus and the SR has been observed via functional magnetic resonance imaging [40].

#### Open issues

The SR model did not receive much attention when it was first proposed. Fortunately, along with the development of multifarious machine learning schemes, the advantages of the SR-based RL have become increasingly noticeable especially in the field of transfer RL [41]. Yet, the research about the SR and its related RL algorithms are still in its infancy, and many questions remain. For example, as for the implementation in transfer RL, the accuracy of the SR model cannot be guaranteed, and further learning is required once the environment changes.

## RL Algorithms Related to Macro-Brain Structure

There is broad consensus that the PFC and BG are 2 main structures related to RL. Specifically, for the PFC, (a) previous neurophysiological experiments have shown that the medial prefrontal cortex (mPFC) can contribute to the regulation of RL parameters such as the learning rate and exploration rate [42]; and (b) entorhinal and ventromedial prefrontal cortex (vmPFC) representations perform a much wider role in generalizing the framework of RL problems [43]. For the BG, although great quantities of computational models of information processing have been developed, the most prominent one is actor–critic of RL [44]. An electrophysiological study in rats shows that neurons in the ventral striatum represent predicted rewards rather than actions, whereas neurons in the dorsal striatum represent actions rather than predicted rewards. This demonstrates the idea that the ventral striatum resembles the critic network and the dorsal striatum resembles the actor network.

On this basis, for brain structures that are related to RL, we concentrate on the PFC and the BG. In the following, we first introduce the PFC-related hierarchical RL (HRL), meta RL (MRL), and prefrontal RL (PRL), followed by a PFC–BG interaction-inspired RL. Also, a summary of these RL algorithms including their macro-brain structure correlations, advantages, and relevant references is presented in Table 3.

**Table 3.** Macro-brain structure-inspired/related RL algorithms

Algorithm	Macro-brain structure correlations	Advantages	References
HRL	Dorsolateral PFC and ACC	Good scalability for problems with high dimensions	[45,46,107]
MRL	PFC	Rapid adaptation for new tasks	[49,108,109]
PRL	PFC	Achieving dynamic arbitration between model-based and model-free	[52–54]
PB-RL	PFC and BG	Suitable for continuous spaces	[55,56]

### Hierarchical RL

#### Description of the algorithm

Conventional RL suffers from a problem of scaling in complex scenarios with high-dimensional space, and its effectiveness degenerates as the scale of the decision-making problem increases. By extending traditional RL to enable temporally abstract actions, HRL can group a series of related low-level actions into hierarchically organized subgoals, and hence dramatically enhances the scalability and learning efficiency [45]. In HRL, the temporally abstract actions are referred to as options, and decision policies would be developed and optimized over these options rather than individual actions. Each option maintains an option-specific prediction error, called pseudoreward prediction error (PPE), and an option ceases when a particular subgoal is achieved. Thus, the principal discrepancy between HRL and RL lies in the way how action is organized. Specifically, in RL, individual actions and the associated RPEs are considered, while in HRL, options that consist of a series of actions and the corresponding PPEs of these options are adopted.

An option *ω* is formally defined as a triple $\left(I_{\omega },\pi_{\omega },\beta_{\omega }\right)$, where $I_{\omega }\subseteq S$ is an initiation set, *π_ω_* is an intra-option policy, and $\beta_{\omega }:S\to \left[0,1\right]$ is a termination function. With this definition, the option-specific Bellman equations, which are consistent with the MDP-specific Bellman equations, are described as follows [46]. For HRL, the action value $Q_{U}:S\times \Omega \times A\to \mathbb{R}$ is the reward of performing an action in the context of a state–option pair, which is expressed as:

$$\begin{array}{c} Q_{U}\left(s,\;\omega ,\;a\right)=r\left(s,\;a\right)+\gamma \sum_{s^{\prime }}‍\mathrm{Pr}\left(s^{\prime }|s,a\right)U\left(\omega ,s^{\prime }\right). \end{array}$$ (23)

The difference between Eqs. 4 and 24 is that due to the existence of the termination function *β_ω_*, the action value *Q_U_*(*s*, *ω*, *a*) needs to consider whether the current option *ω* is terminated or not. This is characterized by the item *U*(*ω*, *s*^′^) that describes the reward of performing *ω* upon reaching a state *s*^′^, which is given by:

$$U\left(\omega ,s^{\prime }\right)=\left[1-\beta_{\omega ,\vartheta }\left(s^{\prime }\right)\right]Q_{\Omega }\left(s^{\prime },\omega \right)+\beta_{\omega ,\vartheta }\left(s^{\prime }\right)V_{\Omega }\left(s^{\prime }\right),$$ (24)

where *β*_*ω*,*ϑ*_ represents the termination function of option *ω* parameterized by *ϑ*.

On this basis, the option-value function is given by:

$$Q_{\Omega }\left(s,\omega \right)=\sum_{a}‍\pi_{\omega ,\kappa }\left(a|s\right)Q_{U}\left(s,\omega ,a\right),$$ (25)

where *π*_*ω*,*κ*_ represents the intra-option policy of option *ω* parameterized by *κ*.

#### PFC correlations of HRL

HRL provides a vigorous computational model for the understanding of abstract action representations, thus indicating the existence of a cognitive hierarchy within PFC. The neural mechanisms underlying the fact of hierarchically organized behavior have been considered to be attributed to the dlPFC, and neuropsychology studies have shown that PPEs positively correlate with the activation of ACC [47]. These results provide evidence for the idea that PFC plays a critical role in encoding subgoals and PPEs to support HRL in the brain.

#### Open issues

A major challenge in HRL is option discovery, that is, how to set the subgoals. Considering the same question in human and other animals, a promising approach, named bottleneck theory, is proposed. By keeping a record of states that occur frequently on paths to final goals, the subgoals that a good solution must pass through can be labeled [48]. Although the study of the neural systems that underlie HRL is just beginning, the recent advanced developments in brain science and neuroscience show brilliant prospects for addressing the challenges in HRL.

### Meta RL

#### Description of the algorithm

**Fig. 4.**
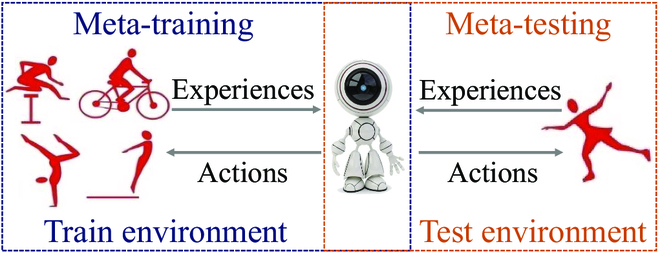
Framework of the meta RL model.

MRL has emerged as a promising strategy for tackling the high sample complexity of RL algorithms, which aims to train agents to learn transferable knowledge that can generalize to new tasks by leveraging prior experiences. As shown in Fig. 4, MRL has 2 stages, i.e., meta-training in the train environment and meta-testing in the test environment. With MRL, the intelligent agent exploits a shared framework among different tasks during the meta-training and enables rapid adaptation to new tasks during the meta-testing from a small number of experiences.

Formally, let W be a space of tasks and *p(w)* be a distribution over tasks w∈W. In MRL, each task is assumed to be an MDP $\langle S,A,T_{w},R_{w}\rangle$ with state space S, action space A, transition distribution *T_w_*, and reward function *R_w_*. All MDPs have the same state and action spaces, while the transition distribution and reward functions are task-specific. During meta-training, the agent executes the tasks that are sampled from *p(w)*, and its goal is to achieve a superior policy for a new task *w*^′^ ∼ *p*(*w*) in meta-testing as efficiently as possible. This can be done in a number of ways, using gradient descent-based methods or recurrent models. For the gradient-based MRL, the meta-training objective can be written as [49]:

$$\begin{array}{c} \left\{\begin{array}{c} \;\max_{\theta }\sum_{w}E_{\pi_{\theta^{\prime }}}\left[\sum_{t}r_{w}\left(s_{t}\right)\right],\\ \theta^{\prime }=\theta +\alpha E_{\pi_{\theta }}\left[\sum_{t}r_{w}\left(s_{t}\right)\nabla_{\theta }\log\pi_{\theta }\left(a_{t}|s_{t}\right)\right]. \end{array}\right. \end{array}$$ (26)

#### PFC correlations of MRL

Much recent work suggests that the PFC not only represents the expected values of actions and states, but also encodes the history of action taking and the correlative rewards [50]. In addition, by stimulating PFC with dopamine, a picture containing 2 mature RL systems is obtained, one based on direct stimulus–response associations and the other leveraging internal representations of task structure. The 2 learning algorithms are independent, and the latter one that can adapt to task environments is generated by regulating the connection weights in the PFC network. The set of variables encoded and the self-contained RL mechanism in the PFC have drawn the conclusion that the PFC constitutes an MRL algorithm whereby one learning algorithm arouses another more efficient learning algorithm for different tasks [51].

#### Open issues

MRL offers a new idea concerning reward-based learning, and some aspects of the MRL need in-depth studies. First, existing gradient-based MRL uses online policy gradients during meta-training. Offline MRL which enables efficient transfer to new tasks without requiring further interaction with the training or testing environments is expected. Besides, in the meta-testing stage, new task learning still involves trade-off between exploration and exploitation. Moreover, the combination between MRL and episodic memory, which may result in smaller inductive biases and higher sample efficiency, is also an attractive direction.

### Prefrontal RL

#### Arbitration mechanism in the PFC

Accumulating evidences have suggested that the brain uses 2 different systems to instruct action selection, that is, a reflexive model-free RL, which is in the form of an RPE that reports the difference between actual and expected reward, and a deliberative model-based RL, which uses a state prediction error (SPE) to learn and improve the structure of the environment. It is found that SPE signals are in the dlPFC and the intraparietal sulcus, whereas RPE signals are in the ventral striatum. The reliabilities of the 2 prediction errors (i.e., RPE and SPE) are encoded by the inferior lateral PFC and frontopolar cortex. A region of ACC is also verified to respond to the difference between the 2 RL models [52].

In addition, for the regions related to value signals of the 2 RL systems, it is revealed that the action value of the model-based one is associated with activity in orbital, mPFC, and parts of ACC, while that of the model-free is associated with activity in dmPFC, dlPFC, and supplementary motor area. Controlled by the output of the arbitrator, a weighted action value that combines the model-based signal and model-free signal is obtained. Substantial correlations with this weighted signal have been found in vmPFC. It is revealed that the model-free values are transmitted to vmPFC in order to combine with the model-based values as a guide [53]. The brain ultimately uses this integrated result to guide behavior.

#### Description of the algorithm

**Fig. 5.**
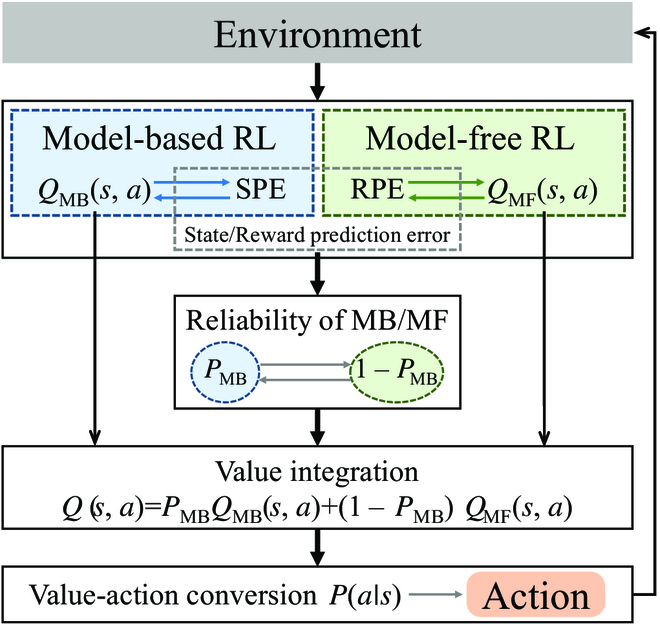
Framework of the prefrontal RL model.

The above evidences support that the PFC is the main brain region involved in arbitration between model-based RL and model-free RL. Motivated by this mechanism, a PRL algorithm is proposed, which is shown in Fig. 5. The action value of the model-based *Q*_MB_(*s*, *a*) and the SPE are updated bilaterally. Similarly, the action values of the model-free *Q*_MF_(*s*, *a*) and the RPE are updated bilaterally. In response to the dynamic environment, learning in the 2 RL systems is mediated by means of the 2 prediction error signals, and then makes inferences about the reliability of model-based and model-free systems according to the relative magnitude of the SPE and RPE. Once the reliability signals are estimated, the probability of a model-based RL *P*_MB_, which characterizes the domination between model-based and model-free, can be determined [54], i.e.,

$$\frac{dP_{\mathrm{MB}}}{dt}=\phi \left(1-P_{\mathrm{MB}}\right)-\psi P_{\mathrm{MB}},$$ (27)

where *ϕ* and *ψ* represent the transition rate MF→MB and MB→MF, respectively. It is seen that the *P*_MB_ is dynamically weighted by the degree of reliability in each RL model.

Then, by integrating MB and MF value estimations, PRL produces an integrated action value *Q*(*s*, *a*),

$$Q\left(s,a\right)=P_{\mathrm{MB}}Q_{\mathrm{MB}}\left(s,a\right)+\left(1-P_{\mathrm{MB}}\right)Q_{\mathrm{MF}}\left(s,a\right),$$ (28)

which is then translated into an action probability *P*(*a*| *s*), according to the taken action.

#### Open issues

For the arbitration mechanism, a number of open questions remain. Despite the findings, how the arbitration-related signals are applied at the neural level to carry out the arbitration computations within PFC is not known clearly. Besides, earlier and present studies about control mechanisms in prefrontal–striatal circuitry raise other biologically reasonable models of the arbitration process, which may go beyond the current algorithmic level. Moreover, to catalyze the developments of such models, a deep understanding of the underlying neural dynamics in these prefrontal areas about the arbitration process is required.

### PFC–BG interaction-inspired RL

It is revealed that the decision-making mechanism in the brain is related to the interaction between the PFC and BG. Specifically, the PFC is regarded to preserve contextual reward information in working memory, and it has a top-down influence on action selection process in the BG [55]. Motivated by the cooperation between the 2 regions in human decision making, a PFC–BG interaction-inspired RL (PB-RL) is proposed, which applies dopamine reward to assess action in the BG and update working memory in the PFC [56].

In the PB-RL, the adopted reward *R*(*s_t_*) integrates the basic reward *r*(*s_t_*) of the current state with evaluation reward *r*_E_(*s_t_*) of the current moment, i.e.,

$$R\left(s_{t}\right)=r\left(s_{t}\right)+\zeta r_{E}\left(s_{t}\right),$$ (29)

where *ζ* is a scale factor.

By maintaining contextual reward information in working memory, the PFC enables flexible and fast decision making. To boost this advantage, by imitating the working memory in the PFC, different TD errors for value function ($\delta_{t}^{V}$) and policy function ($\delta_{t}^{P}$) are considered. In particular, the TD error for policy function is improved by comparing the current reward *R_t_* with the last reward *R*_*t* − 1_, i.e.,

$$\begin{array}{c} \left\{\begin{array}{c} \delta_{t}^{V}=R_{t}+\gamma V\left(s_{t+1}\right)-V\left(s_{t}\right),\\ \delta_{t}^{P}=\left(R_{t}-R_{t-1}\right)+\gamma V\left(s_{t+1}\right)-V\left(s_{t}\right). \end{array}\right. \end{array}$$ (30)

## RL Algorithms Related to Cognitive Function

Attention and episodic memory are 2 crucial cognitive functions for human learning and have been extensively used in machine learning. In this section, we first comprehensively describe the attentional RL (ARL) and the episodic RL (ERL) that are inspired by the cortical attention and episodic memory mechanisms, respectively. Then, we briefly mention other cognitive function-inspired RL algorithms. We also present a summary of these RL algorithms including their cognitive function correlations, advantages, and relevant references in Table 4.

**Table 4.** Cognitive function-inspired/related RL algorithms.

Algorithm	Cognitive function correlations	Advantages	References
ARL	Cortical attention mechanism	Flexible action selection depending on situations	[58,110,111]
ERL	Episodic memory mechanism	Improving sample efficiency	[61,64,112]
Social RL	Positive social interactions	Navigating the social environment	[65]
Optimistic RL	Optimism bias	Robust across different outcome valences	[67]
Unconscious RL	Unconscious metacognition	Untangling the “curse of dimensionality"	[68]

### Attentional RL

#### Attention mechanism

Attention mechanism in the brain enables human to learn and make decisions on only those dimensions that are relevant to the task at hand, and hence can improve performance and speed learning, and simplify generalization to future situations [57]. In neuroscience, the foundation of the attention mechanism is believed to be the cortico–BG–thalamocortical loop. Specifically, striatum receives the prediction signals from the neocortex. Then, the BG outputs an attention signal, which, as a gate, releases the suppression of the thalamic relay cell through the globus pallidus and then mediates the prediction signal [58].

Research in neuroscience suggests a bidirectional support between attention and learning. Specifically, attention constricts learning to the relevant directions of the environment by biasing both value calculation during selection and value updating during learning, while such attentional filters, in turn, would be dynamically adjusted according to the outcomes of ongoing decisions [57,59].

#### Description of the algorithm

**Fig. 6.**
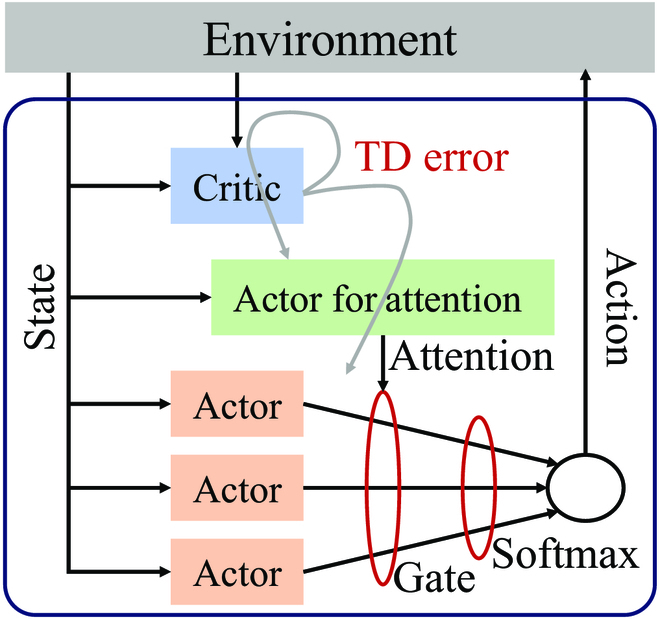
Framework of the attentional RL model.

Attention mechanism has recently emerged as an attractive approach to intelligently selecting contextual information, with successful applications in CV and NLP. Taking inspiration from these tremendous progresses, it is naturally asserted that attention can similarly benefit RL architecture. The enhancement of using attention in RL has been recognized recently. Since the BG has been believed in actor–critic style RL, ARL is intuitively based on the actor–critic model. As shown in Fig. 6, different from the conventional actor–critic model, in ARL, the TD error output by the critic is used for the actor to generate attention signal *A*, and there are multiple actors that would generate different action values for a state. The candidate action values are gated by the attention signal *A*, which corresponds to thalamic relay cells in the brain. The probability of taking action *a* under a state *s* is given by [58]:

$$\pi \left(a|s\right)=\text{softmax}\left(A\right)Q\left(s,a\right).$$ (31)

ARL optimizes the reward function by regulating the flow of prediction signals, which change according to the context. Therefore, unlike traditional RL where the actor has to directly choose an action, ARL enables flexible action selection depending on situations. This capability makes it more competitive in dynamical environments.

#### Open issues

Since ARL has a strong affinity with cognitive function in the brain, it is natural for designing brain-inspired AI models to achieve more human-like decision makings. Besides, the attention mechanism is performed hierarchically in a top-down manner; hence, merging ARL and HRL may build a model that functions as the brain architecture. Moreover, the fact that the attention mechanism for both deep learning and RL is realized in the BG demonstrates that different computational frameworks can be performed on the same brain region, which encourages the integration of multiple learning models in computer science and facilitates the development of brain-inspired AI models.

### Episodic RL

#### Episodic memory

Psychologically, episodic memory is related to long-term, autobiographical snapshots of particular events, which connect many different sensory features of experiences [60]. Physiologically, episodic memory is supported by the hippocampus and associated with medial temporal lobe structures in the brain. Different from the cortical system, which represents statistical summaries of the input distribution, hippocampal learning is thought to be instance-based or nonparametric, which is appropriate for handling sparse, arbitrarily structured, trial-unique data [61].

For episodic memory, the most important idea is that it can provide complete and temporally extended information of the interdependent actions and rewards from individual experiences. Thus, episodic memory enables organisms to (a) approximate value functions in complex state spaces; (b) learn efficiently with quite limited data; and (c) construct long-term connections between action taking and reward function. These capabilities may be the basic reasons why humans can make decisions efficiently and precisely.

#### Description of the algorithm

The increasingly close relationship between episodic memory and RL has illuminated the study of ERL, which offers a possible account for how instance-based processing might subserve reward-driven learning. In ERL, episodic memories are adopted to establish estimations of the state- and action-value functions by implementing a nonparametric approximation. The simplest implementation of ERL is to store historical trajectories. When a familiar state is confronted, retrieve the set of trajectories in that state and then average the obtained rewards to estimate the action value. Formally, we have

$$\begin{array}{c} Q^{\pi }\left(s,a\right)=E_{\pi }\left[\sum_{t=1}^{T}\gamma^{t-1}r_{t}|s,a\right]\approx \frac{1}{N}\sum_{n=1}^{N}R_{n}, \end{array}$$ (32)

where *N* is the number of the retrieved trajectories and *R_n_* is the cumulative discounted return for trajectory *n*.

There are 2 deficiencies of this scheme when applied to more general environments. First, due to the relatively short trajectories, ERL may be myopic and neglects future long-term events. Second, the generalization for problems with complex and continuous state spaces may be inferior.

The first issue can be ameliorated by integrating ERL with the Bellman equation. With a set of trajectories *N*, assuming that an agent starts with action *a* in state *s*_1_ and ends in state *s_T_* within *T* time steps, the value of this state–action pair (*s*_1_, *a*) can be expressed as:

$$Q^{\pi }\left(s_{1},a\right)=\frac{1}{N}\sum_{n=1}^{N}‍\left[R_{n}+\gamma^{T}\sum_{s}‍\mathrm{Pr}\left(s^{\prime }=s|s_{T},\pi \left(s_{T}\right)\right)Q^{\pi }\left(s^{\prime },\pi \left(s^{\prime }\right)\right)\right]$$ (33)

The first term in square brackets in Eq. 33 represents the return from an episode of length *T*, and the second term represents the return after that trace terminated. By combining the 2 terms, the long-term consequences of a finite trajectory are taken into account.

The second problem can be addressed by smoothly interpolating values among episodes. Specifically, the expected return of a trajectory can be approximated by:

$$E_{\pi }\left[\sum_{t=1}^{T}‍\gamma^{t-1}r_{t}|s,a\right]\approx \frac{\sum_{n=1}^{N}‍R_{n}K\left(s,s_{1}^{n}\right)}{\sum_{n=1}^{N}‍K\left(s,s_{1}^{n}\right)},$$ (34)

where $s_{1}^{n}$ represents the initial state of the trajectory stored in the *n*th memory trace, and $K\left(s,s_{1}^{n}\right)$ is the kernel function measuring the similarity between the state *s* and the state $s_{1}^{n}$. In a smooth and real-value state space, a commonly used kernel function is the Gaussian, which is given by:

$$K\left(s,s^{\prime }\right)=\text{exp}\left(-\frac{∥s-s^{\prime }{∥}^{2}}{2\sigma^{2}}\right),$$ (35)

where the parameter *σ*^2^ is used to control the smoothness of the value function approximation.

The developments of episodic memory in psychology and physiology have triggered a growing amount of interest in the research of ERL. Various memory modules enabling episodic memory for RL have been proposed, such as neural episodic control [62] and episodic memory deep Q-networks [63]. On this basis, a short survey on ERL has been presented in the work of Ramani [64]. By giving advantages and disadvantages of some ERL models, it aims to provide insights into how these methods are based on the learning procedures in the brain and promote the idea of usage of episodic memory in RL.

#### Open issues

A challenging issue in contemporary research about RL is the interaction among different learning systems. With the involvement of episodic memory, this issue becomes more prominent. Besides, the tabular-like memories stored as unrelated items in previous work did not reflect the correlations between states. Since associative memory in the hippocampus plays a vital role in human activities, it is expected that the relationship of different experiences may have great inspirations for learning and reasoning.

### Other algorithms

Apart from the attractive attention and memory, other cognitive functions in the brain such as social interactions, optimism bias, and unconscious metacognition also inspire the enhancement of RL. In Jones et al. [65], the authors investigated the behavioral and neural properties of a social RL and suggested that social RL can effectively navigate the social environment. Leveraging emotion analysis [66], the behavioral and neural characterization of an optimistic RL was studied by Lefebvre et al. [67], and an unconscious RL algorithm, which may untangle the “curse of dimensionality" was presented by Cortese et al. [68]. All these exciting frontiers provide key insights into the recent advances in the field of brain-inspired RL.

## Applications

Generally, RL is suitable for sequential decision making, while some nonsequential problems, such as neural network architecture design [69], can also be approached by RL. Therefore, RL shows great potential for a wide range of applications. Games and robotics are 2 classical RL applications; the former are an important testbed for RL/AI, and the latter are meaningful in the era of AI. Besides, NLP and CV enjoy abundant applications of RL in recent years. Note that, since the brain-inspired/related RL algorithms are newly emerging, their applications are quite rare. Thus, the following applications are introduced from a more general perspective of RL.

### Games

Owing to the good or even perfect simulation environment and unlimited data generation, games have been regarded as excellent testbeds for RL/AI technologies [70]. Games can be broadly classified into 3 categories, i.e., board game, card game, and video game. Board games including chess and Go are complete information games with 2 players, while card games like Texas Hold’em Poker and Majiang are incomplete information games with multiple players, and video games may be complete or incomplete information games.

We have witnessed great achievements of human-level or even superhuman performance in games. For board games, a representative example is Go. As a landmark in AI, AlphaGo is the first computer program to defeat a human professional player. The AlphaGo program is an integration of deep neural networks, supervised learning, RL, and Monte Carlo search. Subsequently, AlphaGo Zero further enhances its performance by leveraging a superhuman computer Go program [71], and Alpha Zero inherits the framework of AlphaGo Zero and generalizes it to more domains [72]. For card games, Heads-up Limit Hold’em Poker stands as a typical case, which has been essentially solved by counterfactual regret minimization [73]. For video games, RL applications in various scenarios, such as Minecraft [74] and StarCraft [75], have been studied in the past 5 years.

### Robotics

Robotics is another classic application area for RL, and its motor control and task execution provide abundant validations for developments in RL algorithms. Due to the distinctive features and versatile applications, robotics raises severe challenges to RL, such as multidimensionality, model/environment uncertainty, reward function specification, and sampling issue. As a result, these RL algorithms with ideal assumptions are likely to be a failure in the robotics scenario. To simplify the complexity in the robotic domain, RL has developed tractable approaches by considering several important elements, i.e., representation efficiency, model approximation, and prior-knowledge exploitation [76].

Some remarkable successes are introduced. Rusu et al. [77] adopted a progressive network by reusing low-level visual features to guide high-level policies, which enables multitask transfer. A meta-imitation learning method was presented in Finn et al. [78], with which robots are able to learn more efficiently in complex unstructured environments. With model-free learning, Zhang et al. [79] proposed a deep RL algorithm with successor features for navigation, and Zhu et al. [80] studied a target-driven navigation system for an indoor scenario with the deep RL model. With model-based learning, Finn and Levine [81] developed a method for combining deep action-conditioned prediction with model-predictive control, which enables robots to perform nonprehensile manipulation.

### Natural language processing

NLP refers to learning, understanding, and processing human language content with computer technology [82]. Deep (reinforcement) learning techniques have been permeating into many areas in NLP (such as sequence generation, machine translation, and dialog systems) and help make substantial progresses.

For sequence generation issue, Ranzato et al. [83] proposed a mixed incremental cross-entropy reinforcement by combining REINFORCE and cross-entropy. To improve this scheme, the authors introduced actor–critic methods to train neural networks to generate sequences in the work of Bahdanau et al. [84]. Given the policy of the actor network, the critic network can be utilized to predict the value of an output token. For machine translation issue, Wu et al. [85] conducted a systematic study on training neural machine translation models using RL, and Kang et al. [86] designed a dynamic context selection for document-level neural machine translation via RL. For the dialog systems issue, the current generation is experiencing massive data streams, in which RL would play a vital contribution. A sample efficient actor–critic neural network with experience replay was adopted to design dialog systems with large action spaces in Weisz et al. [87], and a deep RL algorithm with efficient exploration was proposed for multitask dialog systems in Lipton et al. [88].

### Computer vision

CV aims to allow computer machines to obtain an understanding of digital texts, images, and videos [89]. It is used for object recognition and image processing, which can be transformed to decision-making tasks. Recent works have witnessed rapid progresses with the help of (deep) RL to such problems [90].

Object recognition requires algorithms to find bounding boxes for all objects in a given image. For object localization and detection tasks, RL is superior to those approaches with exhaustive search and achieves a good balance between accuracy and efficiency. A tree-structure RL method for sequential object searching to maximize the reward associated with localization accuracy was proposed by Jie et al. [91], and a multiagent RL algorithm for joint object search was deployed by Kong et al. [92]. Image processing involves scene understanding and classification, visual control, motion analysis, and integration with NLP. For this issue, Furuta et al. [93] introduced pixel-wise rewards in RL, which combines RL with convolutional neural networks, thus making it a popular method to solve tasks in image processing. Other applications in CV related to RL include reading maps [94], video analysis [95], and so on.

## Challenges and Opportunities

As stated in the very beginning, conventional RL has many deficiencies for real-world applications. Fortunately, by leveraging knowledge from neurobiology and neuropsychology, brain-inspired/related RL schemes have remedied some defects to a certain degree. To name a few, (a) becaue of the memory mechanism, the sample efficiency of episodic RL is improved; (b) the PFC-related RL algorithms (such as hierarchical RL and PFC–BG interaction-inspired RL) are suitable for large and continuous spaces; and (c) the SR RL and prefrontal RL can strike a good balance between efficiency and flexibility. Despite the tremendous progresses, we are still far from achieving artificial general intelligence, and the way toward this bright prospect is full of challenges as well as opportunities.

• First, it is gradually recognized that many computational challenges currently faced by RL originate from decision-making tasks that the brain handles naturally. Yet, the neural mechanism and brain connectivity in the structure and functional level behind reward-based learning and decision making are not fully understood. Therefore, more detailed knowledge of the neural mechanism, brain connectivity, and cognitive function should be explored to deepen the understanding of how the human brain is controlled and how it learns. 

• Right after the brain mechanism itself, a second critical challenge that lies ahead is to elucidate the corresponding relationship between these findings from brain neuroscience and the elements as well as the framework of RL in computer science. Generally, a functionally sound decision-making process would be implemented in multiple brain areas, and the areas may change with different task demands. In this case, essential questions, such as whether these neural signals in different brain regions are related to the components of RL, and how to represent the complex interaction mechanisms among multiple brain areas by proper RL frameworks and models, need to be answered.

• In addition, even though some correlations and similarities exist between the advanced RL algorithms and the mechanisms that make up human learning ability, human brain’s representation capabilities [96] and control capabilities [97] during learning are still a far reach. The rich and generalizable perception, representations, and control abilities are transferable across tasks. Equipping RL systems with similar representations and sensorimotor abilities of the brain will be a prospective yet challenging work for future research.

• Moreover, since human learning likely involves multiple interacting processes, the integration of multiple advanced RL algorithms would be a promising solution to approaching human-like learning. For example, an episodic-meta RL approach that integrates the MRL and ERL by capitalizing on their complementary benefits was proposed [98]. However, a mathematical theory that can explain all the amazing findings of human brain learning is not available. Therefore, it is crucial for future studies to propose an integrative theory and design computational models to coordinate different types of RL inspired by the brain.

## Conclusion

In this paper, we presented advanced RL in an overview style by summarizing the preliminaries of RL and brain neuroscience, categorizing representative RL algorithms related to brain mechanism from 3 aspects, and discussing diverse applications and potential directions in this amazing field. Briefly, the highly intelligent and efficient decision-making mechanisms in the brain help RL make new remarkable achievements, and the availabilities of biological and psychological engines push the frontiers of RL technology with respect to advanced algorithms and versatile applications. As a consequence, it is expected to embrace the renaissance of RL. Yet, the transfer of ideas between neuroscience and computer science should not be a one-way street. As advanced RL models become more explainable for behavioral data, the paradigm of RL would play a key role in modeling the brain and behavior. Conceivably, such kind of interdisciplinary collaborative efforts provide strong support to close the loop between artificial general intelligence and brain mechanism in both biological and psychological aspects.
